# The implications of internet-based Chinese language courses on online classes

**DOI:** 10.3389/fpsyg.2023.1203136

**Published:** 2024-03-05

**Authors:** Rabnawaz Khan, Weiqing Zhuang

**Affiliations:** ^1^School of Finance and Economics, Fujian University of Technology, Fuzhou, China; ^2^School of Internet Economics and Business, Fujian University of Technology, Fuzhou, China

**Keywords:** COVID-19 pandemic, Chinese language (online-learning), computer-mediated communication, students health, communication mode dominance

## Abstract

Interactionist and social-cultural perspectives on second language acquisition suggest that interactions between teachers and students offer promising avenues for acquiring Chinese as a second language, which the vast majority of international students consider difficult. Computer-mediated communication is far safer than face-to-face encounters during the present pandemic. Three aims are being investigated here. It is important to first analyze the differences between traditional classroom and online learning by different modes, then analyze the various ways teachers use computer-mediated communication, and finally analyze the challenges and opportunities presented by online Chinese as a second language courses using qualitative research methods. Three teachers and 84 students are analyzed statistically in terms of their multimodal interactions, and the quality of their weekly classroom exchanges is assessed through an interpretive analysis of questionnaire data, all in the name of a mixed-methods approach. Particular attention was paid to the challenges of online tutoring for students, the discrepancy between instructor and student understandings, and the use of several teaching strategies with international students. The online classroom environment places unique demands on the quality of student-teacher communication. Different strategies must be used when teaching non-native speakers of Chinese as a second language compared to teaching in a traditional classroom setting.

## 1. Introduction

The popularity of online and other types of distant learning has grown in recent years. There are many benefits to encouraging kids to learn a second language, and doing so is essential for those who hope to reduce cultural barriers in the world. As a result of COVID-19’s impact on the world stage, non-Chinese students studying the language are increasingly receiving their education via online platforms rather than in traditional classroom settings (Yeung and Qiao, 2019). Although there are many advantages to taking classes online, including for students, instructors, and educational institutions as a whole, some teachers have doubts about their reliability and quality, and online learning presents its own unique set of difficulties, especially during times of high anxiety (Ji et al., 2022; Kessler, 2023). We found that faculty can have difficulty transitioning to a more student-centered teaching role, utilizing broader forms of communication, and working to increase student interaction and participation as we reviewed the literature on these issues and challenges in higher education online courses in many different disciplines (Wong and Moorhouse, 2021; Shepperd, 2022). When online courses have substantial enrollments, they can be financially beneficial for institutions. However, these difficulties can be exacerbated when there are many students enrolled in an online course and when the course content seems to call for a lot of student participation, such as in a language class (Darvin, 2023). It is hypothesized that the number of students in a given class is affected by a variety of factors, including pedagogy, the topic or structure of the class, and the required amount of student engagement (Bikowski et al., 2022). However, a significant number of teachers are tasked with leading major sections of classes that are designed to have a high level of student involvement.

In this study, we examine the online Chinese as a Second Language (CSL) classroom from the points of view of teachers who have instructed large numbers of students through the internet (Nam et al., 2023). It is an extension of previous recommendations for additional study to be conducted on the effects of high online class sizes on the process of learning a foreign language and, more especially, on the growth of language instructors. While it is admirable to place a high value on education and the development of one’s self (Li and McLellan, 2021; Yan and Wang, 2022). In this approach, all aspects of education, ranging from the work done by students to the teaching offered by teachers in the classroom, are carried out in a digital setting making use of a broad variety of digital resources. In this way, education is completely transformed into a digital experience (Liang and Teng, 2021). On the other hand, the distribution of online courses is progressively moving toward being done electronically. In light of the critical situation, online education is the only option that can ensure the security of all involved parties (Gong et al., 2018; Cui et al., 2020). But Rebecca Barret-Fox claims that internet education undermines traditional teaching methods. Attempting to have students do the same work they do in class online presents challenges (Wong, 2018). During a pandemic, another difficulty is maintaining and controlling these contacts (online classes) (Kong, 2021). As a result of the current pandemic, university instructors and administrators must accommodate students’ widely distributed locations and time zones (Yang, 2021). In this research study, using cloud-based Zoom software, schools may seamlessly migrate from on-campus to online learning. The application has rapidly gained popularity among non-Chinese speakers studying the language (Tian et al., 2019). With the turn of the millennium, many more schools have started using computers to teach foreign languages (Lu and Révész, 2021; Chen, 2022). In recent years, there has been a significant transition away from a largely textual environment for online language learning toward a multimodal one due to the rise of spoken online communication (Tong and Tsung, 2020).

Accessible through a wide range of mediums, desktop audio/video conferencing and audio-graphic environments that place an emphasis on visual information are now proven effective tools for language learning (e.g., Skype, Elluminate, Flash Meeting, WebCT, NetMeeting, DimDim, etc.). Researchers prior to the rise of the Internet mostly concentrated on spoken and written text, but in multimodal discourse, visual components of text also contributed to the formation of meaning (Hardt et al., 2022; Zhu et al., 2022). In cyberspace, the binaries between modes blur and take on greater nuance. In the age of new media, like Computer-mediated communication (CMC) have access to at least five more channels than the standard verbal, visual, musical, cinematic, and procedural ones. Digital literacy, defined as “the ability to understand and critically apply digital media and communication platforms,” is another set of abilities that students need to succeed in today’s world (Huang et al., 2021; Zhang and Tsung, 2021). While discussing the most readily apparent modalities of Elluminate’s synchronous audio-visual environment (Ashton, 2022). Elluminate is a synchronous audio-graphic environment, so throughout this post we will refer to its most evident modalities, which include: spoken word, pre-loaded text, synchronous writing (text chat), pre-loaded images, synchronously drawn images, and potentially a video image (Xu et al., 2021; Ahern and Biedermann, 2022). Regarding that notably in the pandemic crisis, despite its related challenges, did offer enough possibilities for language educators to experiment with online learning technologies and gain essential knowledge for their future incorporation in language instruction. It is, therefore, crucial for researchers to capture the valuable lessons of this historically unparalleled movement to use technology in language instruction (Rajendram and Shi, 2022). This special issue of the System Journal serves to capture and share these lessons through submissions investigating crucial topics connected to the learning and teaching of languages online (Kruk et al., 2022; Tao and Gao., 2022).

Alongside this, the modes of the classes include procedural components such as yes/no indicators (tick or cross), emoticons (smiley face, perplexed face, clapping hands, thumbs down), and a hand raising symbol that can be used in place of gesture, facial expression, body language, and screen interaction (Smith et al., 2021; Kabilan and Annamalai, 2022). Online language instructors must have expertise in multimodal support in order to give their students a variety of ways to study and learn the language (Chen and Cui, 2022; Nurse-Clarke and Joseph, 2022). Despite the fact that a direct comparison of online and in-person teaching sessions reveals the influence of technological affordances on the patterns of communication and, thus, the different needs of teacher skills and training, there is a dearth of research that examines the impact of video, audio, and text on interaction in an online language classroom (Alarfaj et al., 2022; Beschi et al., 2022; Sak, 2022).

The most significant contribution of this study is an investigation of the influence of online Chinese language courses in various educational, national, and sociocultural contexts, as well as an examination of how teachers and students have responded to these difficulties and proposed solutions (Jiang et al., 2022; Nam et al., 2023). Due to the global nature of the pandemic, many language instructors and their students have been left with little choice but to shift their efforts to online platforms (Klimova, 2021; Gao, 2022). For this anthology, we are especially interested in studies that address pedagogical problems shared by readers in a range of contexts, notably those with fewer resources and those hardest afflicted by the epidemic (Yan and Wang, 2022). As a result, some of the following factors can be used as the basis for strategic decision-making in online classrooms. Traditional barriers to second language (L2) learners generating or co-constructing meaningful dialogs are exacerbated in our case by factors such as first, the relatively low level of language competency of beginning learners, and second, the lack of certain compensating mechanisms in an online setting (Chen, 2022; Hardt et al., 2022; Zhang et al., 2022; Liu et al., 2023). Students need a foundational understanding of both the target language and the technology used to deliver the course material in order to make the most of their time in online language programs (Huang et al., 2021). Some of the compensatory and communication processes that are lost in a computer-mediated classroom are facial expression, body language, gesture, and the assessment of posture. However, participants in an online video or audio conference can use tools like text chat and emoticons to supplement or replace the delegates’ lack of physical proximity to one another (Ashton, 2022).

In light of the current pandemic, this research looks into the methods Chinese teachers are doing to keep their students engaged and motivated in online courses. There are three main goals that this research hopes to accomplish. The study’s goals are to (1) establish the relative benefits of each modality and (2) evaluate the interaction of online classrooms by comparing and contrasting traditional (face-to-face) language lessons with online CSL sessions during the COVID-19 outbreak. Below are some of the most important things we need to know for this research: How does online learning vary from more conventional classroom settings? What different strategies do teachers in a CMC employ in order to aid their students in their educational pursuits? What are the advantages and disadvantages of taking CSL classes online as opposed to the more conventional classroom setting? Furthermore, the first component of this research looks at the differences between online and traditional education in the context of online classrooms. Second, as a result of the epidemic’s rapid spread, schools rushed to transfer their operations online without providing their teachers with proper online classes training on how to utilize these technologies effectively. Therefore, we investigate how various lecturers make use of CMC resources such recorded lectures and online tutorials. The degree to which students actively participate in their online courses is also evaluated. It sets up the framework for this investigation as follows: The literature review is presented in Section 2, data collection is illustrated in Section 3, results are summarized in Section 4, discussed in Section 5, and conclusions and suggestions are drawn in Section 6.

## 2. Literature

The research in this article is grounded in a sociocultural perspective on education. Since language is a product of social interaction, everyone has the right to have his or her native tongue understood and respected (Saha et al., 2022). As a social and dynamic activity, learning a language also involves communicating with others (El-Soussi, 2022). The COVID-19 epidemic has already sparked fear in China, and the situation is dire for the global health community. All Chinese cities are currently adhering to the disaster response procedures that have been established. Institutions of higher education have been closing left and right, especially in developing nations (Rajendram and Shi, 2022). Because of this issue, officials must consider the benefits of either shuttering schools or keeping them open while providing supplementary online services (Chen, 2022).

The purpose of this research was twofold: first, to examine the pros and cons of taking CSL courses online rather than in a traditional classroom environment; and second, to investigate the strategies used by teachers in a CMC to aid their students’ learning (Wong and Moorhouse, 2021; Yu, 2022). Despite students’ beliefs that online language lessons are less successful, the authors find that well-designed online courses can increase student happiness. In addition, other research on the topic of online language learning has concentrated on how students and teachers alike think about and implement advances in online pedagogy such task-based design and genuine language acquisition (Jiang et al., 2022; Nam et al., 2023). Students of different socioeconomic situations, including working adults with aspirations to further their education, are avidly adopting online education. Current changes in education systems around the world may have long-lasting repercussions because it may take years to perfect the shift to online education (Jeon et al., 2022).

TBLT (task-based language teaching) or TBL (task-based learning) is an instructional method that incorporates tasks into the learning process. Consequently, real-world scenarios are utilized as assessment tasks for pupils (Zhang et al., 2019; Zhang and Roberts, 2019; Liang and Teng, 2021). TBL makes language learning more applicable. Computer-mediated communication (CMC) has become a significantly more secure means of human connection, particularly in the education sector, in the present day (Le et al., 2022). The use of collaborative task design, in particular, has been shown to increase students’ perceptions of their own abilities in online language learning environments (Chakraborty et al., 2022). Another study indicated that students working on a group presentation or project established and maintained an online communicative space in which they could share and negotiate ideas. The level of happiness of the students was used as a metric of success in these online courses (Gai et al., 2022). During the course of the pandemic, all parties concerned have praised the choice by many educational institutions to move away from the traditional (face-to-face) mode of teaching in favor of alternatives including web chat, audio, video, and web-conference teaching and learning (Wang et al., 2020; Yang, 2021). The study of online education has come a long way in recent years. When it comes to CSL, however, we cannot make the same claim (Zhu et al., 2022; Watson et al., 2023). During the 2019 winter break, many foreign students studying in China returned home since they were unable to return to China due of the ongoing pandemic (Grammens et al., 2022). In response, many Chinese universities have turned to online platforms to ensure that their international students receive their Chinese language instruction as scheduled (Carrillo and Flores, 2023; Hsiao et al., 2023).

Sociocultural theory is a relatively new branch of psychology that examines the effects of culture on human growth. Vygotsky argues that cultural influences on human development are possible because of the importance of social contact to the maturation of the individual (Mercer and Howe, 2012; Simeon, 2016). Previous research has not considered the implications of online Chinese language courses on the basis of individual teacher and student, thus this study does so (Ward, 2022; Zhang et al., 2022). Students and faculty have been identified as possible interviewees for quantitative studies employing CMC materials like recorded and online lecture, giving this research an advantage over other studies that have adopted differently developed methodologies (Rodgers, 2016; Eccles and Wigfield, 2020). In order to obtain a qualitative perspective, we polled a subgroup of students and three of their instructors via email and Zoom’s smart conferencing. The theory has also gained popularity since the 1990 s1 and has many potential applications in the realms of education, socializing, and even recreation (Shooshtari and Mir, 2014; Simeon, 2016). Lev Vygotsky, a Russian psychologist, proposed that social influences including parents, teachers, classmates, and society at large are crucial in shaping a child’s ability to think abstractly (Sutkin et al., 2022).

### 2.1. Language teaching in an online context

The majority of studies involving teachers and students in a classroom find that classroom contact is an efficient method of language acquisition (Hong et al., 2016). However, thanks to technological advancements, online education is becoming increasingly popular. In recent years, online platforms have become increasingly popular for language instruction (Xue et al., 2013; Cheng and Chiu, 2018). The traditional teacher-student dynamic is giving way to one in which the internet serves as a mediator between educators and their students (Wang et al., 2023). It makes it easier for students and teachers to learn from one another despite physical and temporal distance (Wong, 2018). However, it is more complicated and challenging to produce effective results when engaging in interactive education via a mediator (Hong et al., 2016; Yeung and Qiao, 2019; Khan, 2023a). Under-stimulation, inadequate perceived control over tasks, insufficient attention, and user-unfriendly technology are also mentioned as potential causes of boredom among online CSL learners by the researchers (Yu, 2022). This research enlightens us as to the factors that contribute to the monotony of online language education and prompts us to consider strategies for combating boredom in the classroom (Li, 2020).

Despite the many opportunities for student-teacher engagement that online learning provides, instructors may find it difficult to explicitly characterize the online interactions they have with their students (Wong, 2018). The success of online language courses in helping students improve their listening and speaking skills is astounding. According to our knowledge, only one study has reported on language learners’ readiness for online learning and its correlation with motivation, engagement, learner attitude, and support (Zhang and Hwang, 2023). The authors reveal that language learners show high levels of readiness for online learning, and the predictive power of learner readiness for motivation and engagement highlights the importance for language teachers to promote positive learning attitudes and provide appropriate environmental support to language learners for online learning (Hopp et al., 2022).

### 2.2. Course in virtual Chinese conversation

The teaching of CSL is being practiced in many parts of the world. There have been several books, articles, and studies written about the difficulties associated with teaching and learning Chinese. A positive learning environment and frequent communication with students are essential for success (Yip, 2022). To get the most out of learning a language, it is essential to have productive interactions between students and teachers (Zhang and Dong, 2021). Chinese is both a distinctive and difficult language due to its unique and complex Pinyin script, syntax, characters, and tones. This is why the relationship between teachers and students is so crucial to the success of those studying Chinese (Cui et al., 2020; Ding et al., 2021). Much one-on-one time is required while instructing pupils online. This study is significant for the field of online education because it captures the experiences of foreign students enrolled in Chinese courses at Chinese universities during the 2009 COVID-19 outbreak (Sampietro and Salmerón, 2021; Hsieh et al., 2022; Ross et al., 2022). Studies have not provided sufficient data on student-teacher interactions in online sessions because of the unique characteristics of the Chinese language and the complexity of online learning (Renqiang and Wende, 2022). Five hundred and ten European language students were surveyed, and the results showed that online sessions helped them learn the language more quickly and with less stress (Resnik and Dewaele, 2020). Additionally, students who thrive in language studies are more likely to have high learner autonomy and emotional intelligence. More evidence suggests students who like or take pride in their language learning are more likely to succeed academically (Gao et al., 2022). It showed how a task-based approach to teaching Chinese might be used in a web-based classroom and how both instructors and students may benefit from utilizing a variety of instructional approaches while using asynchronous messaging to communicate (Lyu et al., 2021; Wong et al., 2022). Similarly, our research is centered on the dynamics of student-teacher interaction in virtual CSL classrooms, with an emphasis on the varied approaches taken by different professors (Yu et al., 2022).

## 3. Methodology

The goal of this research is to present a comprehensive picture of how the COVID-19 epidemic has affected Chinese language education (Bikowski et al., 2022; Swanson and Valdois, 2022; Tao and Gao., 2022). In addition, it hopes to use data analysis to examine the interaction process and reveal obstacles specific to this instructional approach. Using questionnaires, this study collected data from both students and instructors. Two questions were addressed in this qualitative study: first, what kind of CSL technique is appropriate for the CMC approach, and second, how typical classrooms’ varying forms were assessed with the support of various teachers’ responses (Zhang, 2021; Zhao and Wang, 2023). Finally, the benefits and drawbacks of doing research over the internet have been weighed from both the participant’s and the interviewer’s points of view. Students and teachers alike have been identified as potential interviewees for quantitative studies using CMC materials like recorded and online lecture (Cheng and Li, 2020). When thinking about that, For the qualitative approach, we conduct in-depth interviews with a sample of 84 students (ST) and three of their teachers (T1, T2, and T3) using email and Zoom’s intelligent conferencing (Gao, 2022; Zhu et al., 2022). We hope to learn from the respondents, among other things, what kinds of challenges they have encountered when taking classes online and what they think of the two distinct delivery methods.

### 3.1. Teaching and learning software

In this study, we relied heavily on the video conferencing app Zoom as our primary method of instruction. Given its ability to provide synchronous video, audio, text chat, facilitate the usage of PPTs during sessions, and provide webinar capabilities (Zheng et al., 2016; Tseng and Zhang, 2020; McDevitt, 2021), especially during the 2009 COVID-19 pandemic, this program became widely employed by a wide range of online educational institutions, growing into a truly enormous teaching platform (Dong et al., 2022; Tao and Xu., 2022). Not only does Zoom provide video recording and meeting rooms, but it also allows you to share applications and desktops (Rajendram and Shi, 2022; Sak, 2022; Yan and Wang, 2022).

### 3.2. Research participants and data collection

Three classes of 28 students each make up the sample size; together, these 84 students and their three Chinese language students had never studied Chinese before. Both teachers and students from Jiangsu University in China were selected for this study. These are introductory Chinese language courses that are 13 weeks long and consist of 39 lessons. In addition, each lesson lasts for 45 min. The average age of the 84 students is 19. According to Table 1, each student is responsible for a group of 28 pupils in one of three distinct classes labeled A, B, and C.

**Table 1:** Number of students.

**Classes**	**NS**	**Countries**
Class A (T1)	28	Russia (9), Ghana (7), United States (2), Pakistan (5), Somalia (3), Egypt (2)
Class B (T2)	28	Ghana (15), Pakistan (3), Yemen (4), South Africa (5), Austria (1)
Class C (T3)	28	Middle East (6), Pakistan (5), India (8), USA (1), Australia (3), Ghana (5)
**Total**	84	

Author computation. T1, T2, and T3 represent teachers 1–3 and NS stands for number of students.

Originally, there were supposed to be three distinct parts to the training. The timeliness and specifics of each phase’s tasks are laid out in Table 2. In the span of the training, three distinct stages were accomplished. Below is the timetable for these stages.

**Table 2:** Course phases and schedule.

**Phases**	**Schedule**
Basic interactions tasks	1st–5th week
Daily conversation	6th–8th week
Professional level interactions	9th–13th week

To make the most of the 45 min allotted for each class. The class progression is established as a set of planned steps. There is a consistent method used across all three stages. Table 3 details these phases and their associated activities, duration, and delivery format (text, video, or audio) (Lu and Révész, 2021; Uludağ et al., 2022). Most of the time in class is spent on activities that put the day’s learning to use. This will guarantee that students have enough time in each class to learn the material and take part in any related games.

**Table 3:** Class stages and activities.

**Stages**	**Activities**	**Time limits**
Pre-task	Warm-up activities	5 min
Main task	Main activities (lessons)	15 min
Reporting stage	Task presentation and class games	25 min

### 3.3. Research technique

Four subgroups (A1-D1) of seven pupils are formed from each teacher’s original group of 28 students. This results in a total of 12 groups. For these subsets, we employ a quantitative and qualitative (mixed-method) strategy by (1) recording and statistically analyzing each educator’s multimodal communications with their classes. (2) The information gleaned from the interviews is examined qualitatively. Each mode begins with an examination of the teachers’ interactions with their classes (Ashton, 2022). The approaches used in each of these modes are dissected in greater detail (Fernández-Batanero et al., 2022; Roose, 2022). Due to its multiple benefits, the mixed-method approach has acquired great traction in the field of research. The mixed-method approach permits researchers to apply both qualitative and quantitative methodologies, thereby combining their respective strengths (Behl et al., 2022). By integrating these two methods, researchers can gain a deeper and more detailed knowledge of the study question. This is because qualitative approaches allow for in-depth investigation of individuals experiences, viewpoints, and emotions, while quantitative methods provide statistical data and generalizability to a larger population. In addition, the mixed-method approach increases the reliability of the results. Using numerous data sources and methods, researchers can triangulate their findings, so enhancing the reliability and validity of their findings. In addition, the mixed-method approach permits researchers to elaborate and clarify the conclusions of one study in light of the findings of another (Ng et al., 2023; Wu et al., 2023). This not only improves the overall analysis, but also enables a more robust interpretation of the data. As a result, during the course of this research, a total of 15 respondents (12 students and 3 teachers) participated in structured interviews that lasted anywhere between 15 and 20 min each. We asked the interviewers more wide, open-ended questions so that we might coax out of them responses that were more specific and in-depth.

### 3.4. Communication modes

We created a tool called Communication Mode Dominance (CMD). By CMD, we mean the frequency with which various modes, such as audio, video, and text, are utilized during a session. Through our analysis, we examined the CMD for both teachers and students (Yang et al., 2021b). We assigned CMD 1 to audio for this investigation, and CMDs 2 and 3 to video and texts, respectively. The CMD is measured and analyzed to assess the interaction between teachers and students in each mode. The book “Developing Chinese” served as the primary course textbook (Zhang et al., 2021; Barrios et al., 2022). A weekly assessment of each student’s overall speech ability (fluency level) was used to determine his/her degree of focus and participation.

### 3.5. Coding

Interviewees’ replies to open-ended questions were used to develop a coding framework based on the most salient themes emerging from the qualitative approach. When assigning codes to interview responses, we based our approach on Colaizzi’s methodology (Sung and Yeh, 2012; Meng et al., 2022; Warner and Diao, 2022). Codes are created by analyzing the responses to 15 observations (interviews) for commonalities in terms of phrases and words (Fenzi et al., 2022; Yasmin, 2022). We arranged for two to three individuals for coding. According to Colaizzi’s method, (i) we listened to the interviews on tape and reviewed the transcripts several times; (ii) extract essential elements and meaningful statements from the transcripts; (iii) code the same elements and statements of the data; (iv) organize the formulated meanings into several clusters of themes; (v) ensure that detailed descriptions were stated and merged for each extracted theme; and (vi) engage in a repeated reading and listening process. According to questionnaires, we utilized the simplest technique for measuring intercoder agreement, which is based on the proportion of decisions. If two codes agree completely on how to code a data set, they will have 100 percent intercoder agreement. Such as agreement subdivided by decision coding. Therefore, we readily comprehend what it signifies for various interviewers, and it is also valuable for monitoring the enhancement of intercoder reliability.

## 4. Results

### 4.1. Multimodal interactions

Zoom’s flexible interface lets students choose from a variety of media for interacting with their instructors and classmates (Abanmy, 2017; Asegid et al., 2023). Any learning goal could be accomplished, for instance, by combining video, audio, and texts. In this study, we take a quantitative method to studying the multimodal interactions between instructors and students (Abedi et al., 2019). The goal here is to get a sense of how often certain modes are actually employed in class.

### 4.2. CMD of each group

**Figure 1:**
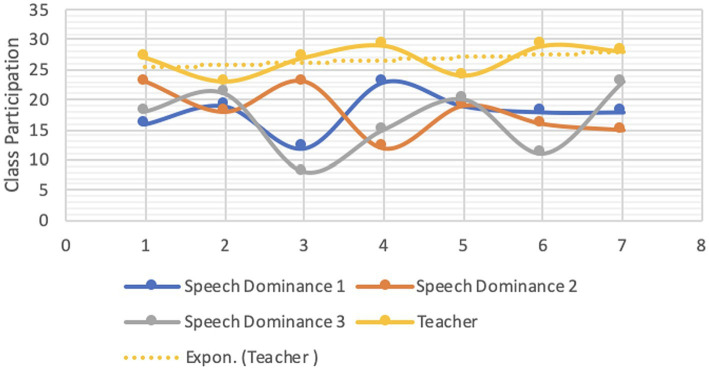
CMD-group A1.

**Figure 2:**
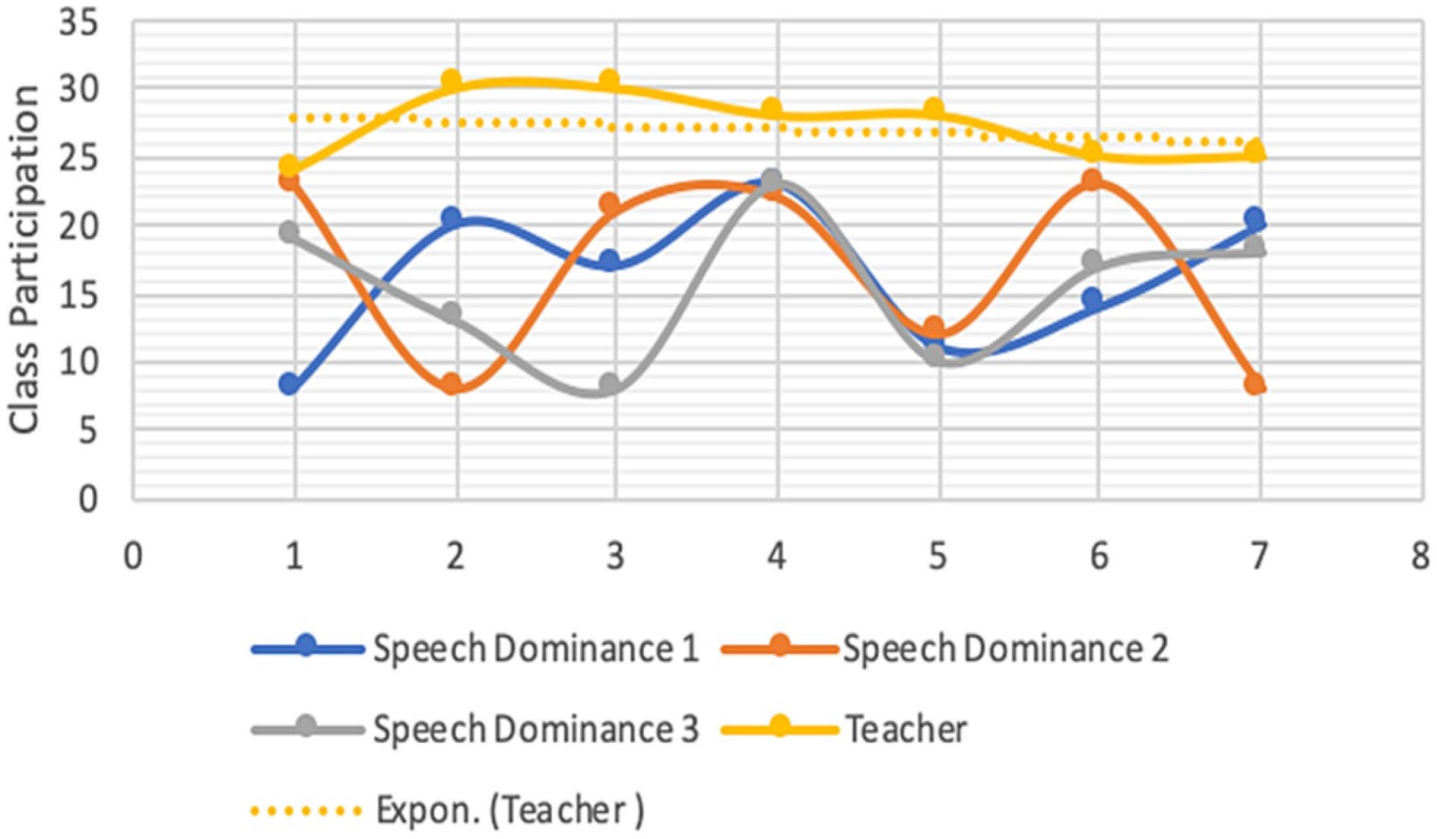
CMD-group B1.

**Figure 3:**
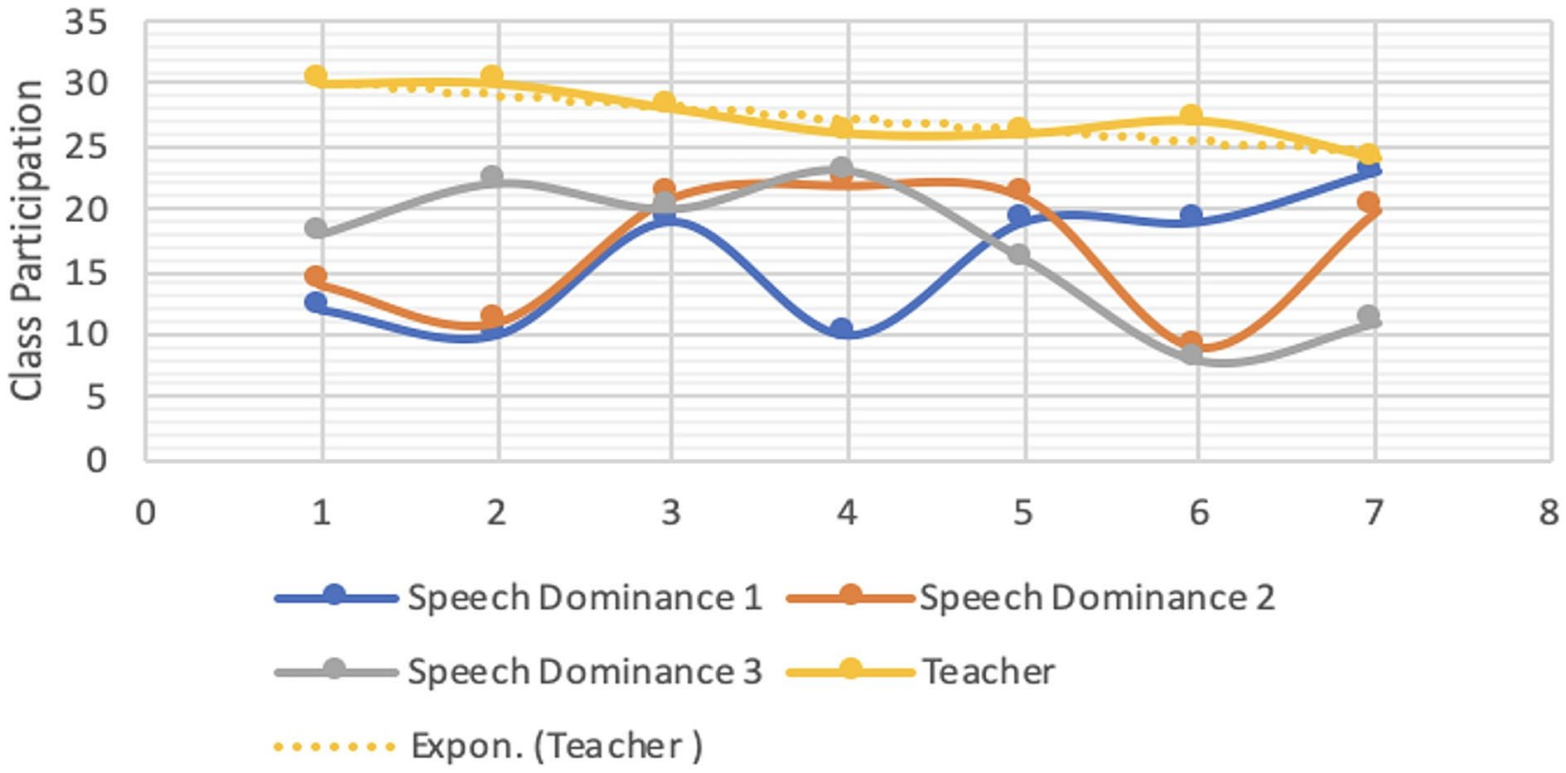
CMD-group C1.

**Figure 4:**
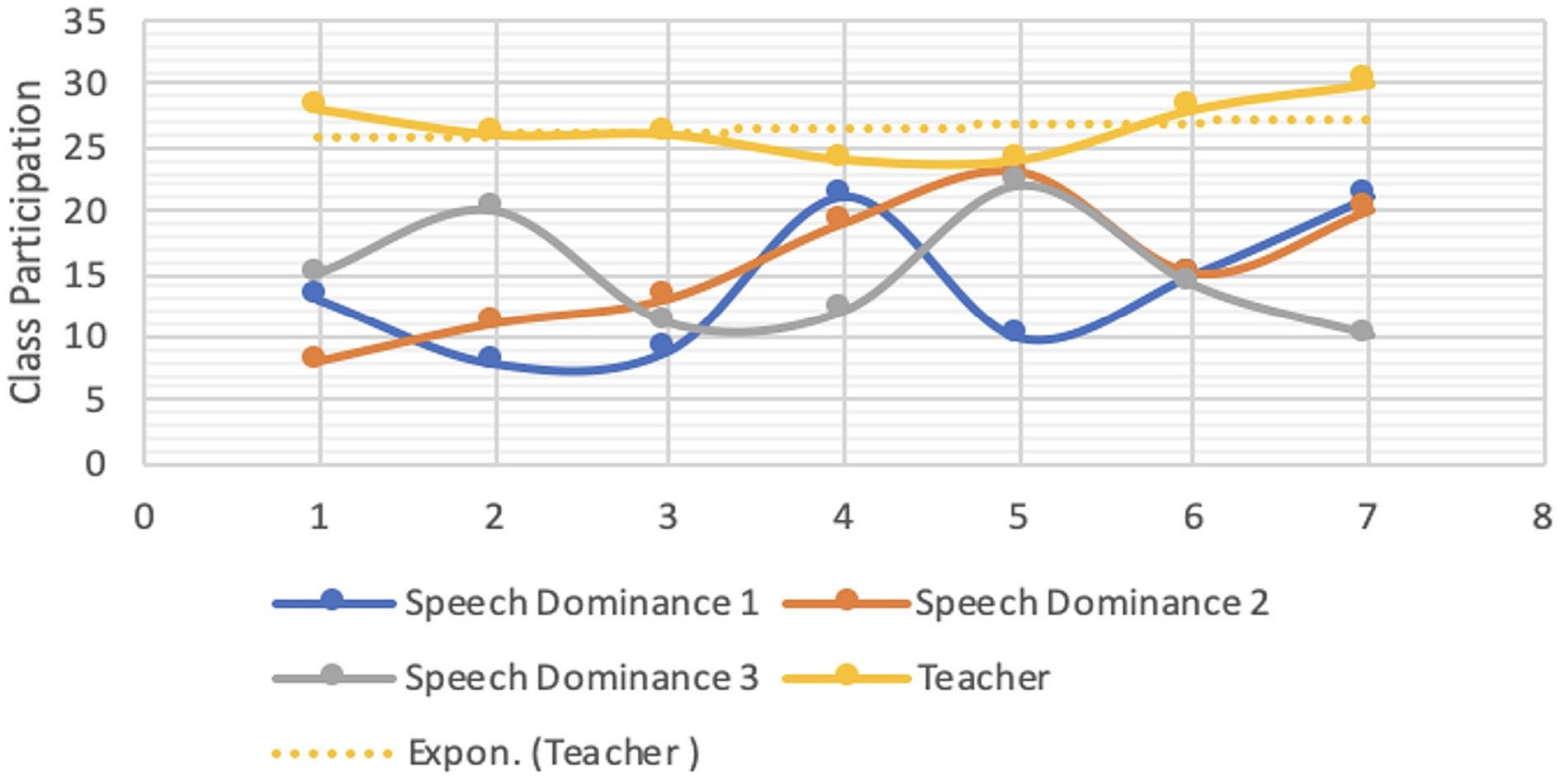
CMD-group D1.

Both students’ and instructors’ CMD were analyzed for each group. Each student’s reading proficiency is measured by their performance in the practice book (Guo and Möllering, 2016; Marino and Cheney, 2022). Figures 1–4 are visual representations of the CMDs, respectively. Figures 1–4 show how each teacher took their initial set of seven students (A–D) and divided them into two smaller groups of three (E–H). Here, we’ll discuss its construction and answer the aforementioned all three questions (Gu and Huang, 2022; Sang, 2023). In response to the first question, one way in which online education differs from traditional classroom settings is that, instead of giving each student a name, teachers assign them a number. Furthermore, the next, ST1-3 was formed from kids in the A group (shared by all three teachers) who had “code 1.” ST 4–6 is comprised of the same three A-group pupils with “code 2″ as ST 2–4. To a similar extent, we treat students with codes 3–7 the same way. Groups B-D use the same method. Groups A1 through D1 are the new designations we have come up with (Helmstaedter et al., 2021). According to Table 4, we finally settled on 7 ST-groups throughout all of A1-D1. The goal was to have a smaller shared sample of children across classrooms (Zhou et al., 2004). The main benefit is that we can evaluate teachers more accurately by looking at how much time they spend with each individual student (Wylde et al., 2019; Chandon et al., 2022). The data in Table 4 is graphically represented in Figures 1–4.

**Table 4:** Weekly average CMD of the three teachers (in minutes).

**Description**	**Group A**1							**Group Average**
**Students**	**ST1-3**	**ST4-6**	**ST7-9**	**ST10-12**	**ST13-15**	**ST16-18**	**ST19-21**	
CMD 1 (Audio)	16	19	12	23	19	18	18	17.857
CMD2 (Video)	23	18	23	12	19	16	15	18.000
CMD3 (Text)	18	21	8	15	20	11	23	16.571
Teachers’ involvement with students	27	23	27	29	24	29	28	26.714
Class total duration	45	45	45	45	45	45	45	45.000
Teaching duration per class	30	30	30	30	30	30	30	30.000
Students’ average (CMD 1, 2, 3)	19.00	19.33	14.33	16.67	19.33	15.00	18.67	17.476
	**Group B**1							
**Students**	**ST22-24**	**ST25-27**	**ST28-30**	**ST31-33**	**ST34-36**	**ST37-39**	**ST40-42**	
CMD 1	8	20	17	23	11	14	20	16.143
CMD 2	23	8	21	22	12	23	8	16.714
CMD 3	19	13	8	23	10	17	18	15.429
Teachers’ involvement with students	24	30	30	28	28	25	25	27.143
Student’s average (CMD 1, 2, 3)	16.67	13.67	15.33	22.67	11.00	18.00	15.33	16.095
	**Group C**1							
**Students**	**ST43-45**	**ST46-48**	**ST49-51**	**ST52-54**	**ST55-57**	**ST58-59**	**ST60-63**	
CMD 1	12	10	19	10	19	19	23	16.000
CMD 2	14	11	21	22	21	9	20	16.857
CMD 3	18	22	20	23	16	8	11	16.857
Teachers’ involvement with students	30	30	28	26	26	27	24	27.286
Student’s average (CMD 1, 2, 3)	14.67	14.33	20.00	18.33	18.67	12.00	18.00	16.571
	**Group D**1							
**Students**	**ST64-66**	**ST67-69**	**ST70-72**	**ST73-75**	**ST76-78**	**ST79-81**	**ST82-84**	
CMD1	13	8	9	21	10	15	21	13.857
CMD2	8	11	13	19	23	15	20	15.571
CMD3	15	20	11	12	22	14	10	14.857
Teachers’ involvements with students	28	26	26	24	24	28	30	26.571
Student’s average (CMD 1, 2, 3)	12	13	11	17.33	18.33	14.67	17	14.762

Authors’ computation. The average time was computed based on individual teacher’s recorded minutes for each student.

### 4.3. Video dominance

Only a tiny percentage of students used webcams or mobile cameras throughout the courses: 18, 16.71, 16.85, and 15.57% in groups A1–D1, respectively (see CMD of Figures 1–4). Only two of the students had their webcams on the entire time, although the rest of the teachers left theirs on the whole time. The interviews conducted shed light on the motivation behind this phenomenon (Guo et al., 2021; Qin and Zeng, 2022). Several students had concerns that they would not be able to participate in the lectures via webcam due to the unreliability and prohibitive cost of mobile data (internet) in their home countries.

In ST1-21, the average times for CMD 1, 2, and 3 were 17.86, 18.07, and 16.57 min, respectively (group A1). Comparatively, the averages for CMD 1, 2, and 3 were 16.14, 16.71, and 15.43 in B1; 16.86, 16.86, and 16.86 in C1; and 13.86, 15.57, and 14.86 in D1 (see Table 3). Among the four groups, group A1 had the greatest average for CMD1 and 2, whereas group C1 had the highest average for CMD3. Group D1 had the worst CMDs overall.

Table 5 displays the schedules for weeks 1, 2, and 3 for each of the three teachers (T1-3). Three classes per week were taught by each instructor, with a 45-min maximum session time. Minutes and seconds are how the educators coordinate their lessons.

**Table 5:** Weekly learning activities.

**Teachers** **and weeks**	**Class**		**Group-Activities (ST)**		**Individual-Activity (T-ST)**	
	**ST**	**T-ST**	**Same**	**Different**	**Same**	**Different**
T1-Week 1	14′45”	15′15”	4′25”	3′00”	3′20”	4′15”
T1-Week 2	16′34”	13′26”	3′30”	4′15”	2′00”	5′15”
T1-Week 3	11′55”	18′05”	1′15”	4′10”	5′05”	4′30”
T2-Week 1	5′41”	24′19”	6′40”	3′73”	1′22”	3′26”
T2-Week 2	11′21”	18′39”	0′20”	4′30”	5′05”	5′05”
T2-Week 3	8′31”	21′29”	3′30”	3′95”	3′12”	4′23”
T3-Week 1	7′05”	22′55”	4′45”	4′55”	2′15”	3′45”
T3-Week 2	10′25”	19′35”	1′75”	4′50”	4′19”	4′16”
T3-Week 3	4′06”	25′54”	7′95”	3′56”	0′25”	2′84”

Authors’ computations. ‘and” represent minutes and seconds. ST, student, T-ST, teacher and student.

The total class time of 45 min is broken up into three parts (class, group, and individual activities). When people talk about “class,” they are referring to the time when lessons are being given. This is broken down even further into ST (student review) and TT (teacher introduction and instruction) for the day’s topic (T-ST). There will be time for both teamwork and solo pursuits throughout the first 15 min. Every educator appeared to give each component of the lessons their full attention. Activities for both groups and individuals were planned in such a way that the same or different tasks might be assigned at different times.

The graph demonstrates that it takes significantly more time to complete a task in a group than it does to complete it alone. It is possible that this is because the group will need to discuss many options before coming to a decision (Huang et al., 2022; Qin and Zeng, 2022). Conversations also show that activities in which members of a group are all working on the same thing take significantly longer than those in which they are all working on separate things (Chu et al., 2015; Loh et al., 2018; Sun and Teng, 2021).

**Figure 5:**
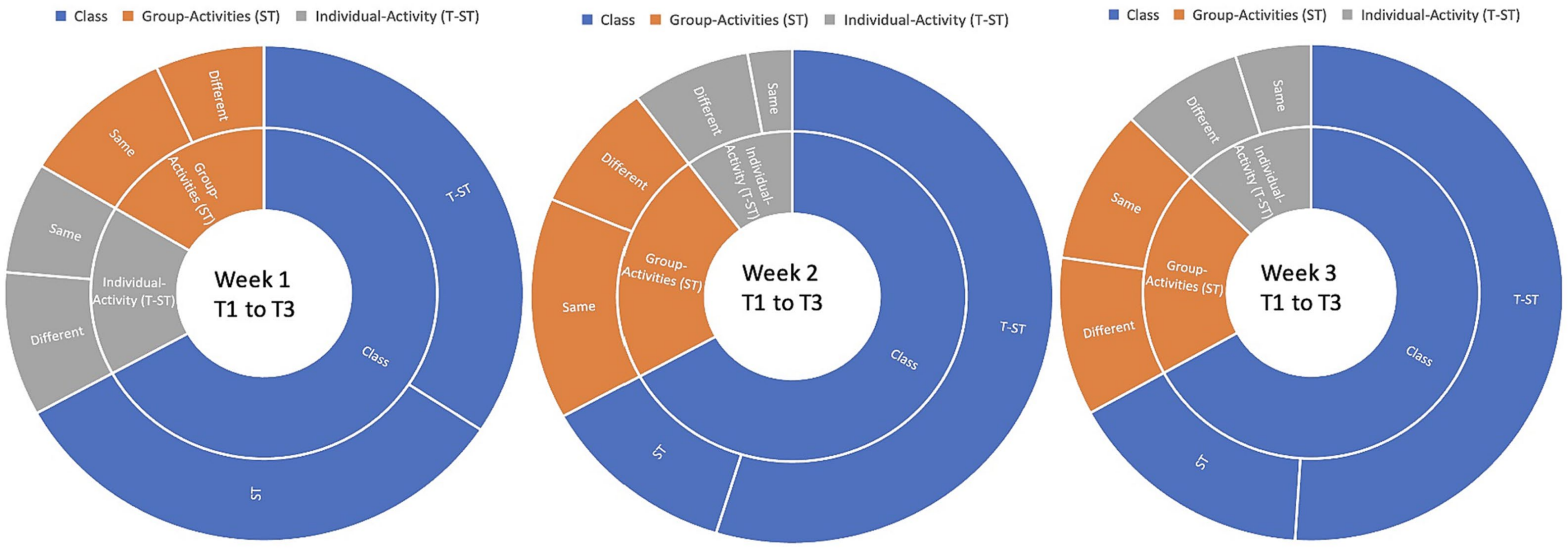
T1–T3.

A further takeaway from Figure 5 is the fact that educators used a wide range of strategies to engage their students. T3 spent 7 min, 43 s on task-based practice and 2 min, 5 s on games during week 1, whereas T3 spent 5 min, 45 s on task-based practice and 1 s on games during the same week. In addition, during the first week of school, T3 spent 67.75 percent of class time on mechanical practice and 5 percent on games. In addition, T2 spent 46.7% of its time during the third week on mechanical practice and 30.5% on task-based activities. Teachers check in with their students’ health at the start of each lesson by asking if they have taken the online health assessment. Jiangsu University developed a daily online health survey to collect data on the well-being of its student body.

**Figure 6:**
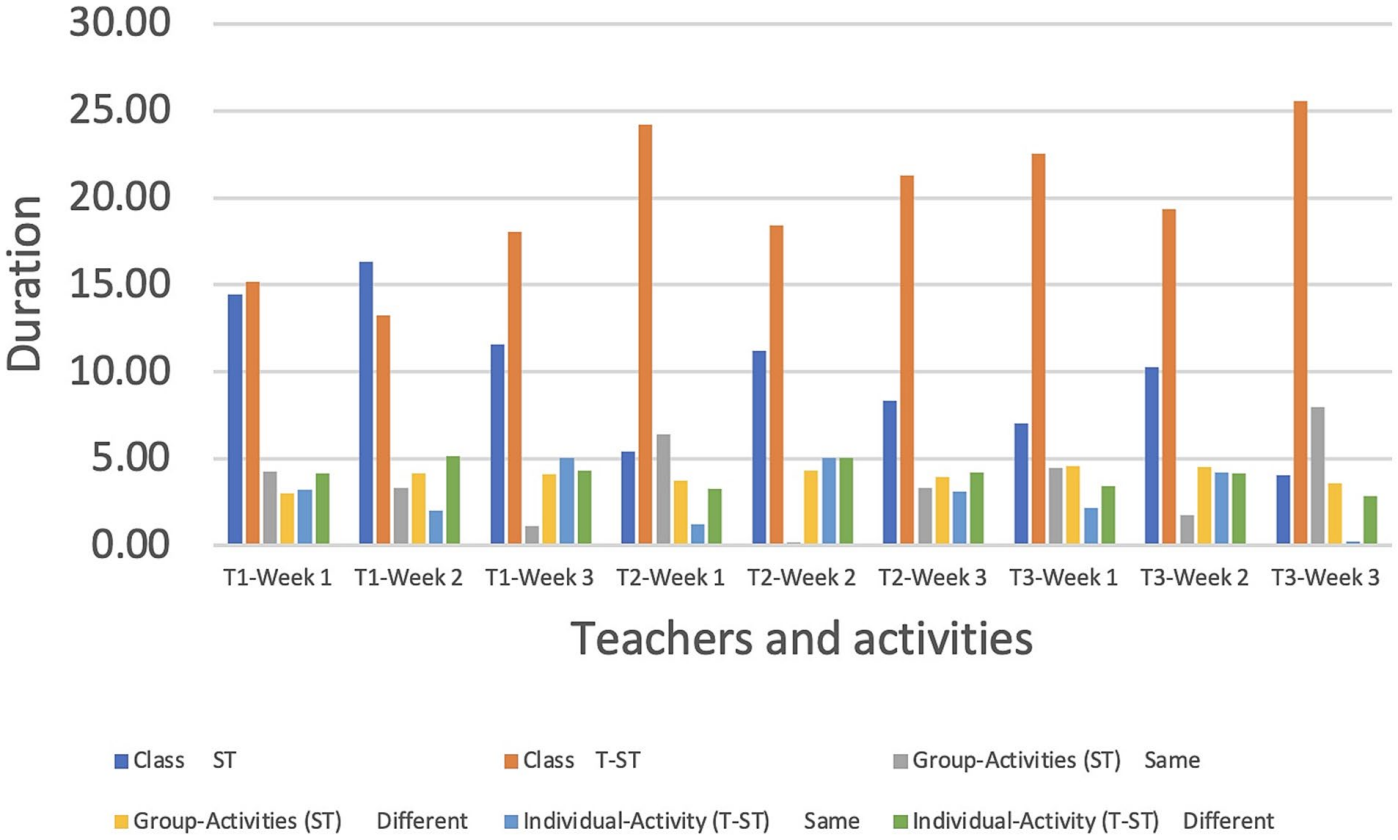
T-ST activities in weeks 1–3.

The class discussion shed light on the types of CMC methods asked about in the second question. In-class activity (T-ST) is better suited to the CMC method of teaching since it encourages instructors to use a variety of strategies and to include their students in a wide range of projects and activities. The separate classes received the same lectures from the same lecturers. ST and T-ST exercises were performed in a variety of ways during each session. In Figure 6, we can see the class, group, and individual activities that occurred in Weeks 1, 2, and 3 from each teacher and their respective presentation style, which may be the same or vary from week to week. All teachers’ weekly class performance and methods are graphed for easy comparison. Comparing weeks 1 and 3, T3 spent 25 min and 54 s (25′ 54″) on T-ST exercises, while T1 only spent 13 min and 26 s (13′ 26″) on the same task. It is more difficult for teachers to keep track of their students’ progress in online classrooms if they do not all focus on the same tasks at once. CSL takes a higher level of commitment from students and teachers than is usually necessary when learning a new language (Zhang et al., 2020; An and Thomas, 2021). Because of this, educators are tasked with devising methods to pique students’ interests throughout class.

### 4.4. Network stability

In 11 interviews, 73.3% of respondents said that a stable network is essential for CMC. Both instructors and students reported spending significant time dealing with technological concerns (i.e., the internet) before and during class time. Therefore, the curriculum had to be modified by the educators. Again, the interviews demonstrated that students in low-connectivity locations would be unable to maximize their productivity.

Teachers’ ability to give each student equal attention was hampered by the fact that they could not see all of them at once, despite the fact that they could engage with each other using Zoom’s smart functions like raising hands and sharing stickers (An and Thomas, 2021; Sampietro and Salmerón, 2021). Additionally, teachers were unable to see their students’ physical appearance during online classes, making it challenging for them to gauge factors like facial expressions and attentiveness (Ji et al., 2022). Even though most students said that the internet and other outside sources did not have a big effect on how well they did in school, it was nice to see that both teachers and students were generally optimistic.

### 4.5. Interactive content of online classes

Because of the resources and environments made available by online education platforms, effective and efficient education is possible. The use of synchronous online education at Jiangsu University has resulted in the creation of an atmosphere that is quite comparable to that of a conventional classroom (Kruk et al., 2022). Due to time limits, both teachers and students must be present at the appointed meeting time (Wang et al., 2021). The schedules for weeks 1–3 of all three online professors’ classes are presented in Table 6. In all, this study’s online lesson consists of a warm-up, instruction, practice, and conclusion exercise.

**Table 6:** Weekly class activities.

**Teachers and weeks**	**Pre-task (Warm up)**		**Main task** **(Teaching)**			**Practicing**			**Wrap up**
	**Call-att.**	**Daily-con**	**Words**	**Grammar**	**Text**	**Mechanical practice**	**Task-based practice**	**Class games**	**Summarize homework**
T1-Week 1	1′15”	3′45”	5′26”	4′18”	5′16”	10′24”	5′16”	4′20”	5′00”
T1-Week 2	2′00”	3′00”	8′25”	4′35”	2′00”	12′35”	5′25”	2′00”	5′00”
T1-Week 3	3′36”	1′24”	7′19”	4′21”	3′20”	9′34”	6′01”	4′25”	5′00”
T2-Week 1	2′45”	2′15”	8′24”	4′10”	2′26”	10′12”	7′43”	2′05”	5′00”
T2-Week 2	4′20”	0′40”	8′25”	4′20”	2′15”	13′11”	5′24”	1′25”	5′00”
T2-Week 3	3′20”	1′40”	10′00”	3′35”	1′25”	12′15”	5′45”	2′00”	5′00”
T3-Week 1	3′25”	1′35”	8′35”	5′17”	1′08”	13′55”	5′45”	1′00”	5′00”
T3-Week 2	1′45”	3′15”	5′30”	5′20”	4′10”	14′25”	3′10”	2′25”	5′00”
T3-Week 3	1′50”	3′10”	7′53”	4′52”	2′55”	10′11”	7′25”	2′24”	5′00”

Call-att. represents calling of attendance; Daily-con represents daily conversation.

**Figure 7:**
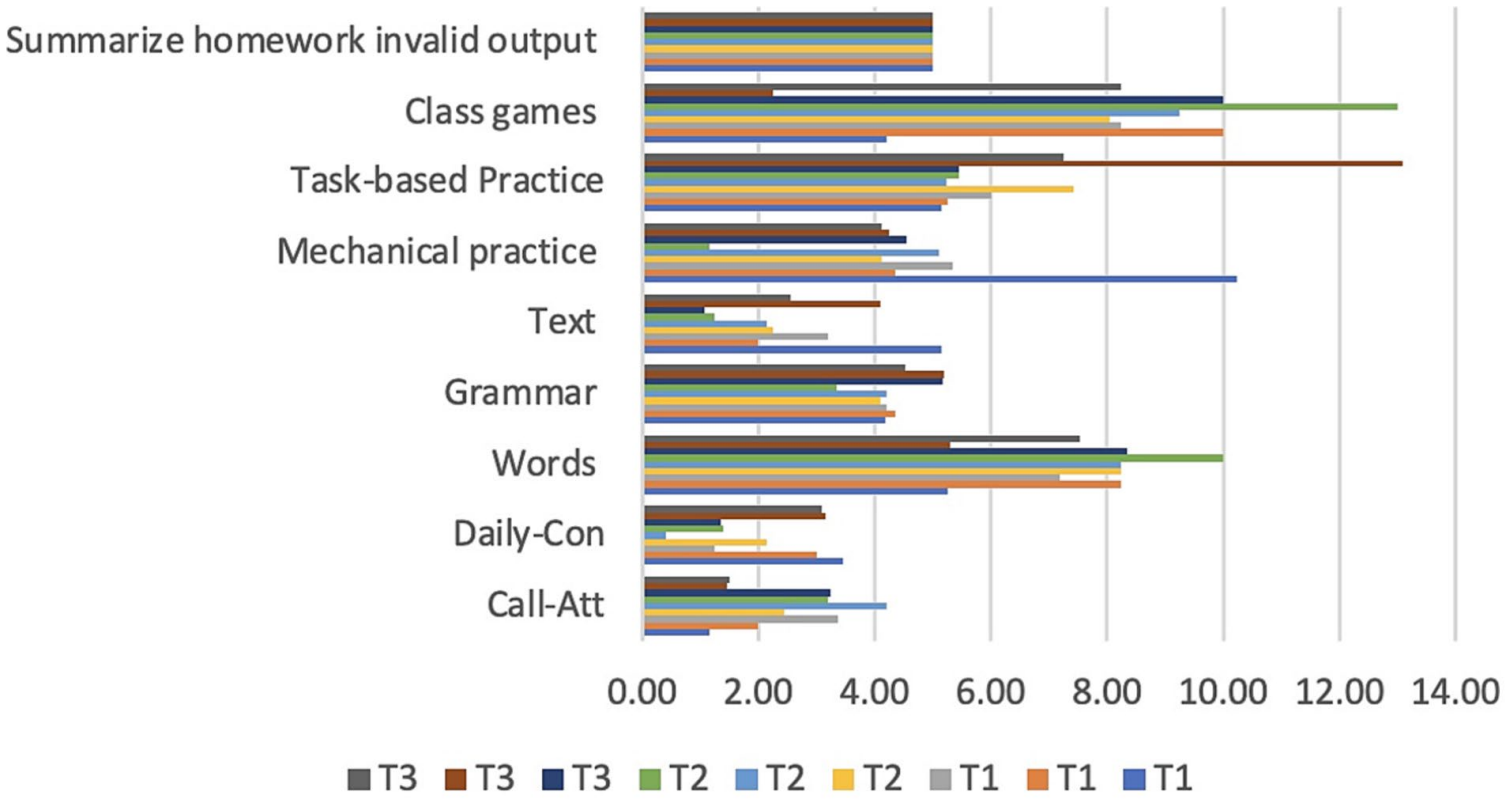
Weekly class activities.

Based on the weekly plan provided, it appears that teachers set aside roughly 5 min each day for warm-up exercises. Before the day’s activities begin, this time allows teachers to take roll and have a little chat with each kid. It is a wonderful opportunity for teachers to keep up with their pupils on a personal as well as intellectual level (Yan and Wang, 2022). Additionally, the primary lessons in each class were given 15 min of time (Xu et al., 2021). The main task for the task was broken up into three smaller sections. Lessons were delivered using a variety of methods by the educators. The difference between T1’s time spent on vocabulary development in week 1 (5 min and 26 s) and T2’s time spent on the same task in week 3 (10 min and 00 s) is stark. In this study, it was determined that introducing new vocabulary took the greatest time, followed by grammatical concepts, and finally text (Figure 7). Twenty minutes were allotted for the third section of each lecture. During this period, students will engage in games and other task-based activities designed to review the day’s content. Due to the limitations of human contact, class games at CMC are less engaging than in a typical classroom, so more time is spent on mechanical skills at this stage. It is customary to spend the final 5 min of class going over the day’s subject and homework.

### 4.6. Training of language skills

Oral communication skills are essential for success in CSL. Regarding the third question, T1, T2, and T3 have discussed the benefits and drawbacks of the CSL classes. It is well-established that language proficiency coincides with proficiency in other areas. First, from observing the three T1, T2, and T3 courses, we can tell that teachers spend a total of 5 min at most on teaching certain Chinese characters (texts) (Table 6). Vocabulary and text are both relevant to reading (Moeiniasl et al., 2022). Reading is followed by discussion questions, which can be led by either the teacher or the students. The most challenging aspect of education (Gao, 2022), whether it be received online or in a traditional classroom setting, is the development of one’s oral communication abilities (Tao and Gao., 2022). Over the course of the study period, T1 played listening materials (audio recordings) while the other teachers bolstered students’ listening skills through conversation.

## 5. Discussion

The findings appear to indicate that both educators and those being educated in Chinese online should be prepared for a wide variety of potential difficulties (Zhao and Kuhl, 2022). Preparation is the key to success when delivering an online lesson, as teachers must be able to communicate their goals to their students without ambiguity. In order to draw pedagogical conclusions, we focused on situations where there was a disconnect between what was intended and what was understood, as well as on coping mechanisms and communication breakdowns (Gai et al., 2022; Yu et al., 2023). Because of the inherently social aspect of teaching and learning, we suggest CMD tactics like in different groups a self-inventory of their teaching practices, based on these strategies, to assist attendings improve their intraoperative teaching (Rodgers et al., 2020). Analyze the many ways in which teachers employ computer-mediated communication, and then evaluate the benefits and drawbacks of teaching Chinese as a second language online, all through the lens of qualitative research (Frederick et al., 2022b; Sheng et al., 2022). In the spirit of combining qualitative and quantitative methods, we conduct a statistical analysis of the multimodal interactions between three educators and 84 pupils, and we evaluate the quality of their weekly classroom exchanges using an interpretation of questionnaire data (Frederick et al., 2022a).

The theoretical foundation of this research is Sociocultural Theory (SCT), which is based on the idea that the purpose of forming relationships between students and teachers in the classroom is to help students succeed academically (Han et al., 2023; Moradkhani and Mansouri, 2023). The connections facilitate communication with others and participation in lessons. According to the findings above, the primary purpose of the research was to determine the pros and cons of taking CSL courses in a traditional classroom format. As for the qualitative method, we looked at 84 students (ST) and their professors (*n* = 3) (T1, T2, and T3). Among other things, we intend to find out from respondents what kinds of difficulties they have encountered when attending classes online and how they feel about the two different delivery systems (Lai, 2023; Ren and Zhu, 2023). The second goal was to learn more about how CMC educators facilitate their students’ learning. Thus, we employed this theory in the second phase of our inquiry into various ways to determine the sociocultural features and the significance of these aspects in terms of the relationship between students and teachers in conventional classroom settings as opposed to online classes (Tian et al., 2023).

Students’ concerns about online tutoring, the gaps in understanding between teachers and their students, and the effectiveness of different approaches to instruction with international pupils were all given special focus (Khan, 2021; Rodgers et al., 2022). Furthermore, we zeroed in on three areas in particular: the difficulties associated with teaching Chinese students online, the gap between what the tutor intends and what the student perceives, and the utilization of many modalities when dealing with students from other countries (Kong and Khan, 2019; Liu and Kinginger, 2021; Tulviste and Tamm, 2021). The above results indicated that weekly average of CMD of different teachers in which CMD2 results have highly instinctually affect comparatively CMD1 and CMD2 (Säljö, 2010). Particular attention was paid to the challenges of online tutoring for students, the discrepancy between instructor and student understandings, and the use of several teaching strategies with international students (Frederick et al., 2022b; Montoya et al., 2022). One of the challenges of online education is raising the bar for student-teacher interaction. Different strategies must be used when teaching non-native speakers of Chinese as a second language compared to teaching in a traditional classroom setting (Crooks et al., 2020).

All new language learners encounter difficulties, but those who are just starting out in Chinese experience challenges that are unique among those encountered by speakers of other languages. Adding written resources to oral activities is also frequent when teaching a foreign language (Khan, 2022; Zhai and Wibowo, 2022). But what unique difficulties (linguistically, technologically, and culturally) does online education for Chinese present? The fact that Chinese uses strange, non-alphabetic characters reduces the reliability of this framework (Hopp et al., 2022). Furthermore, there is very little overlapping terminology. Although few Chinese language students would be able to cope with this, certain approaches of communicative language training argue for the exclusive use of the target language in the classroom (Zhang et al., 2019). Therefore, non-English language teachers usually utilize English in their sessions for full beginners, even though the purpose is to introduce more target language communication through other media (Zhang et al., 2020). Here, we examine the most effective methods for getting from A to B in online courses taught by a wide range of specialists (Li and McLellan, 2021).

It seems challenging to teach a language to novices online, despite the many benefits of multimodal online teaching, such as the choices to cater to reluctant students and for different learner types, give feedback in different modalities, etc. (Mansouri et al., 2021; Najmuddin et al., 2022), and run the classroom more efficiently (Khan and Zheng, 2021; Tu, 2021; Yang et al., 2021a; Chung and Fisher, 2022). Adding mode and media switching to an existing heavy burden is possible. While text chat’s scaffolding for speech may be helpful for certain kids, the abundance of possibilities may be overwhelming for others (Yang et al., 2021b; Brice et al., 2022; Khan, 2023b). It is especially true in a widening class, where students have varying majors and levels of “proximal development,” that learning occurs in a social environment (in which the learner gets), as argued by Vygotsky’s SCT. Therefore, it is crucial that teachers grasp this concept and offer supplementary aid to students whose progress is slower than the rest of the class. The authors of this study found that students’ engagement with the material increased when it was tailored to their own culture and daily life. The use of visual aids like videos and animations was designed to improve students’ grasp of the material and their ability to retain it (Li et al., 2023). The course was revamped to include a variety of student activities (from mini-projects to presentations). Student responses show that increasing the relevance of course material to students’ real-world experiences increases their motivation and leads them to act more like active learners (Chen and Wang, 2023). In addition, increasing the variety of classroom activities can better accommodate children at varying developmental stages, allowing all learners to be stretched and pushed to their full potential (Chung and Fisher, 2022; Zhang, 2023).

## 6. Conclusion and recommendation

According to the evidence presented above, it is extremely difficult to successfully teach a course using CMC. Due mostly to connectivity concerns, the majority of non-Chinese students did not receive platform training prior to the start of class. Such pupils required additional time to adjust to their new classroom. Students may have internalized a bias toward online education as a result of this situation. Students view their professors as authoritative characters in the classroom so that they can anticipate upcoming events and lessen any fear they may be experiencing. Teachers must be well-versed in both pedagogical software and best practices for online instruction in order to confidently address potential classroom difficulties. Therefore, we have less wasted time and improved information flow.

Improving the level of student-teacher communication is another obstacle that online courses must overcome. Online instruction of elementary CLS for non-native speakers demands a different approach than classroom instruction. Therefore, lecturers need to adapt their methods to include the most interesting and engaging components possible. Teachers and students alike often resort to the “avoidance approach” in the face of confusion. For questions that can be answered with a single word, written communication may be preferable to oral exchange. Even if there are no recorded lectures available for students to review, they should still be allowed to take advantage of supplementary instruction. Students who, for various reasons (the internet, for example), did not fully grasp a concept or course can now catch up in time for their exams. Students can learn a lot from one another in a group setting. Teachers can have students form smaller groups and give each group a specific topic to discuss or a specified number of sentences to write. Students are more motivated to work hard when they have a clear goal in mind. Teachers should move from group to group frequently to listen in on discussions and push for increased usage of Chinese in all classroom activities. The state-of-the-art video conferencing for businesses offered by Zoom is a safe, cloud-based platform for mobile phone-based audio, video, chat, and webinars. The recording function in Zoom makes it easy to capture classroom instruction for the purpose of subsequent review. Instructors in online courses should amass sufficient materials to deliver effectively. In addition to avoiding PowerPoint presentations and audio resources, students should continue speaking Chinese outside of the classroom.

This study only comprised 84 students and 3 faculty members from China’s Jiangsu University, and they all used the online Zoom platform. Only three of the 13 weeks of class time were used in our analyses. Because of it is broad applicability, more people may gain from it. Different people at different proficiency levels will respond differently to an online Chinese course. Although this study lays a good groundwork for future research, it is only relevant to novice CSL learners.

However, there are two caveats to this study that need to be mentioned. In the first place, all of the participants in this study were adults from economically disadvantaged nations like South Africa or Ghana, thus they might not be representative of CSL learners more generally, especially those from wealthier countries or younger age groups. As a result, it is not possible to extrapolate these results to the CSL learning population as a whole. In the future, larger research might include a wider range of CSL students from different ethnic and cultural backgrounds. Second, the short time period addressed by the study may not be indicative of the complete picture of emotional growth among CSL students. Future studies may choose to track CSL students’ moods for longer to determine how they factor into the students’ development. Thus, second-language learners’ FLE factor structure may vary by group. Considering that students regard FLE components differently is vital to their interest in learning a foreign language.

## Data availability statement

The original contributions presented in the study are included in the article/supplementary material, further inquiries can be directed to the corresponding author.

## Author contributions

RK’s contributions included the acquisition, analysis, drafting, editing, and verification of the integrity of the data analysis. In addition, RK developed the study strategy and interpreted the outcomes that were anticipated. Conceived of, planned, and executed the revision of this manuscript, as well as performed thorough editing on the manuscript. WZ focused on conceptualization of data, acquisition, modification, and manipulation of data, interpretation of data, drafting, integrity of data and providing approval for publication. All authors listed have made a substantial, direct, and intellectual contribution to the work and approved it for publication.
